# Racial and Ethnic Variation in Survival in Early-Onset Colorectal Cancer

**DOI:** 10.1001/jamanetworkopen.2024.46820

**Published:** 2024-11-22

**Authors:** Joshua Demb, Scarlett L. Gomez, Alison J. Canchola, Alexander Qian, James D. Murphy, Robert A. Winn, Matthew P. Banegas, Samir Gupta, Maria Elena Martinez

**Affiliations:** 1 Division of Gastroenterology, Department of Internal Medicine, University of California, San Diego, La Jolla; 2 Moores Cancer Center, University of California, San Diego, La Jolla; 3 Department of Epidemiology and Biostatistics, University of California, San Francisco; 4 Helen Diller Family Comprehensive Cancer Center, University of California, San Francisco; 5 Department of Radiation Medicine and Applied Sciences, University of California, San Diego, School of Medicine, La Jolla; 6 Massey Cancer Center, Department of Medicine, Virginia Commonwealth University, Richmond; 7 Herbert Wertheim School of Public Health and Human Longevity Science, University of California, San Diego, La Jolla

## Abstract

**Importance:**

Rates of early-onset (before 50 years of age) colorectal cancer (EOCRC) are increasing, with notable differences across racial and ethnic groups. Limited data are available on EOCRC-related mortality differences when disaggregating racial and ethnic groups.

**Objective:**

To investigate racial and ethnic differences in EOCRC mortality, including disaggregation of Asian American populations separately, including Native Hawaiian or Other Pacific Islander populations and specific Asian American groups, and to quantify the contribution of clinical and sociodemographic factors accounting for these differences.

**Design, Setting, and Participants:**

This population-based cohort study included California Cancer Registry data for individuals aged 18 to 49 years with EOCRC between January 1, 2000, to December 31, 2019. Median follow-up was 4.2 (IQR, 1.6-10.0) years. The data analysis was conducted between July 1, 2021, and September 30, 2024.

**Exposure:**

Race and ethnicity defined as Asian American (and 7 disaggregated subgroups), Hispanic, Native Hawaiian or Other Pacific Islander, non-Hispanic American Indian or Alaska Native, non-Hispanic Black, and non-Hispanic White.

**Main Outcomes and Measures:**

Cox proportional hazards regression models were used to measure association between race and ethnicity and CRC mortality risk, yielding adjusted hazard ratios (AHRs) and 95% CIs. Associations of sociodemographic, health system, and clinical factors with differences in mortality by racial and ethnic minority group were assessed using sequential modeling.

**Results:**

There were 22 834 individuals diagnosed with EOCRC between 2000 and 2019 (12 215 [53.5%] male; median age, 44 [IQR, 39-47] years). Racial and ethnic identity included 3544 (15.5%) Asian American, 6889 (30.2%) Hispanic, 135 (0.6%) Native Hawaiian or Other Pacific Islander, 125 (0.5%) non-Hispanic American Indian or Alaska Native, 1668 (7.3%) non-Hispanic Black, and 10 473 (45.9%) non-Hispanic White individuals. Compared with non-Hispanic White individuals, higher EOCRC mortality was found for Native Hawaiian or Other Pacific Islander (AHR, 1.34; 95% CI, 1.01-1.76) and non-Hispanic Black (AHR, 1.18; 95% CI, 1.07-1.29) individuals. Disaggregation of Asian American ethnic groups revealed notable heterogeneity, but no single group had increased EOCRC mortality risk after full adjustment for covariates. For Hispanic individuals, there was higher EOCRC mortality (AHR, 1.15 [95% CI, 1.08-1.22]) with the base model (adjustment for age, sex, and tumor characteristics), but the association disappeared once neighborhood socioeconomic status was added to the base model (AHR, 1.00 [95% CI, 0.94-1.06]). Similarly, there was higher EOCRC mortality among Southeast Asian individuals with the base model (AHR, 1.17 [95% CI, 1.03-1.34], but that association disappeared with the addition of insurance status to the model (AHR, 1.10 [95% CI, 0.96-1.25]).

**Conclusions and Relevance:**

In this cohort study, racial and ethnic disparities in EOCRC mortality were evident, with the highest burden among Native Hawaiian or Other Pacific Islander and non-Hispanic Black individuals. These results provide evidence of the role of social determinants of health in explaining these differences.

## Key Points

**Question:**

Are there racial and ethnic differences in mortality in early-onset colorectal cancer (EOCRC)?

**Findings:**

In this cohort study of 22 834 participants, higher EOCRC mortality was shown for Hispanic, Native Hawaiian or Other Pacific Islander, non-Hispanic Black, and Southeast Asian adults compared with non-Hispanic White adults. Neighborhood socioeconomic status and insurance status attenuated the differences in EOCRC mortality when factored into the analysis.

**Meaning:**

These results suggest that racial and ethnic disparities in EOCRC mortality are evident, and findings of potential roles of social determinants of health underscore the need to better understand these disparities.

## Teaser

This cohort study examines race- and ethnicity-based differences in mortality risk among patients with early-onset colorectal cancer, notably disaggregated Asian American and Native Hawaiian or Other Pacific Islander groups, and assesses the association of individual- and neighborhood-level social determinants of health with mortality risk.

## Introduction

Colorectal cancer (CRC) is the second leading cause of cancer-related death in the US.^1,2^ The incidence and mortality of CRC in adults younger than 50 years—hereinafter referred to as early-onset colorectal cancer (EOCRC)—has been increasing in the US since the 1980s, with a notable recent 1.9% annual increase in incidence and a 1.2% increase in mortality observed from 2011 to 2019.^2,3,4^ By 2030, it is estimated that over 10% of all colon cancers and 23% of all rectal cancers will be diagnosed in patients younger than 50 years.^2,5^

While numerous studies have identified a higher EOCRC burden among American Indian or Alaska Native, Hispanic, and non-Hispanic Black individuals,^2,6,7,8^ data on whether EOCRC incidence and mortality disproportionately affect Asian American and Native Hawaiian or Other Pacific Islander adults are limited, with one study showing unchanged EOCRC incidence between 2001 and 2016^9^ and another showing no significant CRC-related mortality disparity compared with non-Hispanic White adults.^10^ However, it is well recognized that the Asian American and Native Hawaiian or Other Pacific Islander racial category comprises a heterogeneous group, including with respect to social determinants of health.^11^ Few studies have provided risk estimates for disaggregated Asian American and Native Hawaiian or Other Pacific Islander individuals. Further, studies of disparities in EOCRC survival have not considered the impact of social determinants of health above and beyond that of individual-level clinical and sociodemographic factors, despite increasing knowledge of the importance of upstream structural and social factors.^12,13^ These evidence gaps limit our ability to design and tailor clinical and public health interventions to reach those with the greatest EOCRC-related mortality burden.

Our present study leveraged data from the population-based California Cancer Registry (CCR) to examine race- and ethnicity-based differences in mortality risk among patients with EOCRC, notably disaggregated Asian American groups. In addition, we assessed the relative contribution of individual- and neighborhood-level social determinants of health as contributors to mortality risk across racial and ethnic groups.

## Methods

### Study Patients

CRC cases were identified using data from the CCR, which contains demographic, clinical, treatment, and outcome information for patients with cancer who were residents of California at diagnosis. We included all patients aged 18 to 49 years with a first primary invasive CRC between January 1, 2000, and December 31, 2019 (n = 27 167). Full inclusion and exclusion criteria are detailed in eMethods in Supplement 1, yielding a total study population of 22 834 patients. Reporting on study design, analyses, and results follow guidelines outlined by the Strengthening the Reporting of Observational Studies in Epidemiology (STROBE) statement, specific to cohort studies. The study was approved by the institutional review board at the University of California, San Francisco, who waived the need for informed consent owing to use of deidentified registry data.

### Data Acquisition and Variables

CCR data, mostly derived from the individual’s medical record, were used to obtain diagnosis date, age at diagnosis, race and ethnicity, sex, marital status, individual-level insurance status, American Joint Committee on Cancer tumor stage, tumor grade, tumor location, tumor size, guideline-concordant treatment, and whether the individual received cancer care at a National Cancer Institute–designated cancer center (eTable 1 in Supplement 1). Neighborhood-level social determinants of health included geocoded census block group of residential address at diagnosis, neighborhood socioeconomic status (SES), and urban-rural status.

Race and ethnicity were classified as Asian American (including Chinese, Filipino, Japanese, Korean, South Asian, Southeast Asian, and Other Asian), Hispanic, Native Hawaiian or Other Pacific Islander, non-Hispanic American Indian or Alaska Native, non-Hispanic Black, or non-Hispanic White according to medical records and CCR classification system, supplemented by the North American Association of Central Cancer Registries’ identification algorithms for Asian American and Hispanic population groups using factors such as race and ethnicity, birthplace, and names.^14^ Additional details regarding insurance status, guideline-concordant treatment, and residential urban-rural status are provided in the eMethods in Supplement 1. We used a common multicomponent measure of neighborhood SES based on individuals’ residential census block group at diagnosis, categorized into quintiles based on the statewide distribution.^15^ This measure, an adaptation of a neighborhood SES measure developed by Yost et al^16^ for use with American Community Survey data, incorporated 2000 US Census (for cases diagnosed in 2005 and earlier) and the 2007-2011 American Community Survey data (for cases diagnosed in 2006 and forward) on educational attainment, occupation, unemployment, household income, poverty, rent, and house values. Block groups are census-defined administrative units conceptualized to best represent neighborhoods, sized between 600 and 3000 residents.

### Follow-Up

The CCR followed up individuals for vital status, from linkage with vital records and hospital follow-up, to December 31, 2019. Follow-up time was calculated as the number of days from diagnosis until the earliest of date of CRC-specific death, date of death from another or unknown cause, date of last known contact, or December 31, 2019. From the study population of 22 834 patients, there were 6883 CRC-specific deaths (30.1%), 979 other deaths (4.3%), and 216 deaths of unknown cause (0.9%) observed during the study period. The median follow-up time was 4.2 (IQR, 1.6-10.0) years overall, 2.0 (IQR, 1.0-3.6) years for those who died of CRC, and 7.0 (IQR, 2.6-12.7) years for those who were alive at the end of follow-up.

### Statistical Analysis

Data were analyzed between July 1, 2021, and September 30, 2024. To estimate the association of race and ethnicity with risk of CRC-specific death, hazard ratios (HRs) and 95% CIs were calculated using multivariable Cox regression. For models with an outcome of CRC-specific death, individuals who died of other causes were censored at the date of death and those with an unknown cause of death were excluded. Models using Fine and Gray estimation^17^ accounted for the competing risk of death from non-CRC causes. As a secondary analysis, models were calculated with an outcome of all-cause mortality. A minimally adjusted model was adjusted for clustering by block group, using a sandwich estimator of the covariance structure that accounts for intracluster dependence. A base model was additionally adjusted for age, year of diagnosis, sex, and tumor size, with underlying stratification by American Joint Committee on Cancer stage, tumor grade, and tumor location. A fully adjusted model additionally included marital status, insurance status, National Cancer Institute–designated cancer center, neighborhood SES, and neighborhood urban-rural status, with additional underlying stratification by guideline-concordant treatment. Age and year of diagnosis were included in models as continuous variables with a linear and a quadratic term, and all other variables were treated as categorical. These models were run with the Asian American ethnic groups combined and then with results shown separately for each ethnic group. Given the recent changes to the CRC screening guidelines, additional age-stratified sensitivity analyses were conducted to compare findings among adults aged 18 to 44 vs 45 to 49 years.

To assess the whether social and neighborhood factors altered associations beween EOCRC mortality in specific racial and ethnic groups after accounting for age, sex, and tumor characteristics in a base model, sequential models were run, in which social and neighborhood factors were entered into the model one at a time based on the order in which the parameter estimate for a particular racial or ethnic group changed, from most decreased to most increased, when each factor was added individually. Resultant change in effect for each race and ethnicity compared with non-Hispanic White individuals due to adjustment could be interpreted as evidence of confounding or mediation. Additional information regarding the statistical modeling can be found in eMethods in Supplement 1. We provided the adjusted HRs (AHRs) and 95% CIs for each sequential model. Results were shown for racial and ethnic groups that had a statistically significantly (2-sided *P* < .05) higher risk than non-Hispanic White individuals in the base model. Analyses were performed using SAS, version 9.4 (SAS Institute Inc).

## Results

The study population included 22 834 patients with EOCRC (Table 1). This included 3544 Asian American (15.5%; median follow-up, 4.2 [IQR, 1.7-10.0] years), 6889 Hispanic (30.2%; median follow-up, 3.3 [IQR, 1.3-8.0] years), 135 Native Hawaiian or Other Pacific Islander (0.6%; median follow-up, 2.6 [IQR, 1.1-5.5] years), 125 non-Hispanic American Indian or Alaska Native (0.5%; median follow-up, 4.3 [IQR, 2.1-8.9] years), 1668 non-Hispanic Black (7.3%; median follow-up, 3.7 [IQR, 1.3-9.6] years), and 10 473 non-Hispanic White (45.9%; median follow-up, 5.1 [IQR, 1.9-11.2] years) individuals. There was a lower proportion of female compared with male patients in all racial and ethnic groups except for Native Hawaiian or Other Pacific Islander; overall the study population included 10 610 female (46.5%) and 12 215 male (53.5%) individuals, with a median age of 44 (IQR, 39-47) years. Compared with non-Hispanic White individuals, Hispanic, Native Hawaiian or Other Pacific Islander, and non-Hispanic Black individuals had higher proportions of individuals residing in low SES neighborhoods. Among Asian American, Hispanic, non-Hispanic American Indian or Alaska Native, and non-Hispanic White individuals, most tumors were in the rectum. The largest proportion of cancers was found in the proximal colon in non-Hispanic Black individuals and in the distal colon in Native Hawaiian or Other Pacific Islander individuals.

**Table 1.** Sociodemographic and Clinical Characteristics of Patients With CRC Stratified by Race and Ethnicity in the California Cancer Registry, 2000 to 2019.

Characteristic	Race and ethnicity group, No. (%) of patients^a^						
	All (N = 22 834)	Asian American (n = 3544)	Hispanic (n = 6889)	Native Hawaiian or Other Pacific Islander (n = 135)	Non-Hispanic American Indian or Alaska Native (n = 125)	Non-Hispanic Black (n = 1668)	Non-Hispanic White (n = 10 473)
Age, y							
18-39	5911 (25.9)	873 (24.6)	2203 (32.0)	48 (35.6)	36 (28.8)	372 (22.3)	2379 (22.7)
40-44	6104 (26.7)	979 (27.6)	1848 (26.8)	29 (21.5)	36 (28.8)	445 (26.7)	2767 (26.4)
45-49	10 819 (47.4)	1692 (47.7)	2838 (41.2)	58 (43.0)	53 (42.4)	851 (51.0)	5327 (50.9)
Year of diagnosis							
2000-2004	4924 (21.6)	734 (20.7)	1175 (17.1)	22 (16.3)	22 (17.6)	399 (23.9)	2572 (24.6)
2005-2009	5722 (25.1)	900 (25.4)	1542 (22.4)	29 (21.5)	36 (28.8)	479 (28.7)	2736 (26.1)
2010-2014	5889 (25.8)	901 (25.4)	1871 (27.2)	34 (25.2)	34 (27.2)	403 (24.2)	2646 (25.3)
2015-2019	6299 (27.6)	1009 (28.5)	2301 (33.4)	50 (37.0)	33 (26.4)	387 (23.2)	2519 (24.1)
Sex							
Female	10 610 (46.5)	1752 (49.4)	3181 (46.2)	70 (51.9)	56 (44.8)	780 (46.8)	4771 (45.6)
Male	12 215 (53.5)	1792 (50.6)	3705 (53.8)	65 (48.1)	68 (54.4)	887 (53.2)	5698 (54.4)
Transgender or other	9 (0.04)	0	<5	0	<5	<5	<5
AJCC stage							
I	3502 (15.3)	505 (14.2)	919 (13.3)	17 (12.6)	19 (15.2)	242 (14.5)	1800 (17.2)
II	4797 (21.0)	740 (20.9)	1542 (22.4)	20 (14.8)	25 (20.0)	336 (20.1)	2134 (20.4)
III	7375 (32.3)	1175 (33.2)	2199 (31.9)	48 (35.6)	34 (27.2)	499 (29.9)	3420 (32.7)
IV	5932 (26.0)	942 (26.6)	1828 (26.5)	43 (31.9)	38 (30.4)	483 (29.0)	2598 (24.8)
Unknown	1228 (5.4)	182 (5.1)	401 (5.8)	7 (5.2)	9 (7.2)	108 (6.5)	521 (5.0)
Tumor location							
Proximal colon	5623 (24.6)	792 (22.3)	1728 (25.1)	29 (21.5)	29 (23.2)	607 (36.4)	2438 (23.3)
Distal colon	7654 (33.5)	1285 (36.3)	2319 (33.7)	54 (40.0)	41 (32.8)	532 (31.9)	3423 (32.7)
Rectum	9041 (39.6)	1397 (39.4)	2664 (38.7)	48 (35.6)	52 (41.6)	485 (29.1)	4395 (42.0)
Other	516 (2.3)	70 (2.0)	178 (2.6)	<5	<5	44 (2.6)	217 (2.1)
Tumor grade							
1	1860 (8.1)	245 (6.9)	613 (8.9)	8 (5.9)	9 (7.2)	122 (7.3)	863 (8.2)
2	14 810 (64.9)	2265 (63.9)	4456 (64.7)	86 (63.7)	84 (67.2)	1101 (66.0)	6818 (65.1)
3	3586 (15.7)	656 (18.5)	1005 (14.6)	18 (13.3)	24 (19.2)	246 (14.7)	1637 (15.6)
4	280 (1.2)	39 (1.1)	88 (1.3)	<5	0	21 (1.3)	128 (1.2)
Unknown	2298 (10.1)	339 (9.6)	727 (10.6)	19 (14.1)	8 (6.4)	178 (10.7)	1027 (9.8)
Tumor size, cm							
0.1-2.9	3069 (13.4)	501 (14.1)	756 (11.0)	7 (5.2)	16 (12.8)	163 (9.8)	1626 (15.5)
3.0-3.9	2712 (11.9)	471 (13.3)	692 (10.0)	19 (14.1)	14 (11.2)	176 (10.6)	1340 (12.8)
4.0-4.9	3288 (14.4)	568 (16.0)	946 (13.7)	14 (10.4)	14 (11.2)	251 (15.0)	1495 (14.3)
≥5.0	9139 (40.0)	1308 (36.9)	3050 (44.3)	64 (47.4)	52 (41.6)	726 (43.5)	3939 (37.6)
Missing or other	4626 (20.3)	696 (19.6)	1445 (21.0)	31 (23.0)	29 (23.2)	352 (21.1)	2073 (19.8)
Marital status							
Married	13 454 (58.9)	2434 (68.7)	3988 (57.9)	87 (64.4)	54 (43.2)	652 (39.1)	6239 (59.6)
Not married	8618 (37.7)	989 (27.9)	2647 (38.4)	45 (33.3)	65 (52.0)	946 (56.7)	3926 (37.5)
Unknown	762 (3.3)	121 (3.4)	254 (3.7)	<5	6 (4.8)	70 (4.2)	308 (2.9)
Insurance status							
None or self-pay	809 (3.5)	105 (3.0)	378 (5.5)	0	<5	79 (4.7)	245 (2.3)
Medicaid only	3807 (16.7)	447 (12.6)	1884 (27.3)	28 (20.7)	30 (24.0)	375 (22.5)	1043 (10.0)
Medicare	859 (3.8)	88 (2.5)	228 (3.3)	<5	<5	115 (6.9)	422 (4.0)
Military or other public	765 (3.4)	104 (2.9)	282 (4.1)	5 (3.7)	<5	86 (5.2)	284 (2.7)
Private	16 034 (70.2)	2725 (76.9)	3895 (56.5)	98 (72.6)	85 (68.0)	980 (58.8)	8251 (78.8)
Unknown	560 (2.5)	75 (2.1)	222 (3.2)	0	<5	33 (2.0)	228 (2.2)
Diagnosis and/or treatment at NCI-designated cancer center							
No	18 892 (82.7)	2867 (80.9)	5778 (83.9)	113 (83.7)	99 (79.2)	1462 (87.6)	8573 (81.9)
Yes	3942 (17.3)	677 (19.1)	1111 (16.1)	22 (16.3)	26 (20.8)	206 (12.4)	1900 (18.1)
Guideline-concordant treatment							
Yes	17 705 (77.5)	2763 (78.0)	5262 (76.4)	106 (78.5)	97 (77.6)	1222 (73.3)	8255 (78.8)
No	3428 (15.0)	523 (14.8)	1088 (15.8)	16 (11.9)	15 (12.0)	298 (17.9)	1488 (14.2)
Unknown	1701 (7.4)	258 (7.3)	539 (7.8)	13 (9.6)	13 (10.4)	148 (8.9)	730 (7.0)
Neighborhood SES quintile							
1st (Lowest)	4060 (17.8)	323 (9.1)	2412 (35.0)	16 (11.9)	23 (18.4)	436 (26.1)	850 (8.1)
2nd	4402 (19.3)	560 (15.8)	1821 (26.4)	29 (21.5)	36 (28.8)	389 (23.3)	1567 (15.0)
3rd	4565 (20.0)	657 (18.5)	1287 (18.7)	38 (28.1)	29 (23.2)	376 (22.5)	2178 (20.8)
4th	4951 (21.7)	902 (25.5)	958 (13.9)	30 (22.2)	22 (17.6)	314 (18.8)	2725 (26.0)
5th (Highest)	4856 (21.3)	1102 (31.1)	411 (6.0)	22 (16.3)	15 (12.0)	153 (9.2)	3153 (30.1)
Neighborhood urban-rural status							
Rural	1127 (4.9)	43 (1.2)	248 (3.6)	6 (4.4)	15 (12.0)	25 (1.5)	790 (7.5)
Town	865 (3.8)	31 (0.9)	347 (5.0)	7 (5.2)	13 (10.4)	16 (1.0)	451 (4.3)
City	5936 (26.0)	564 (15.9)	1751 (25.4)	21 (15.6)	45 (36.0)	353 (21.2)	3202 (30.6)
Suburb	11 278 (49.4)	2237 (63.1)	3011 (43.7)	76 (56.3)	46 (36.8)	880 (52.8)	5028 (48.0)

Abbreviations: CRC, colorectal cancer; NCI, National Cancer Institute; SES, socioeconomic status.

^a^ Percentages have been rounded and may not total 100. Cell numbers are suppressed when less than 5.

### Findings Across Racial and Ethnic Groups

Compared with non-Hispanic White individuals, Native Hawaiian or Other Pacific Islander individuals had a higher likelihood of EOCRC mortality after adjusting for clustering by block group (AHR, 1.69; 95% CI, 1.27-2.23); for age, sex, and tumor characteristics (AHR, 1.42; 95% CI, 1.07-1.87); and individual- and neighborhood-level social determinants of health (AHR, 1.34; 95% CI, 1.01-1.76) (Table 2). Non-Hispanic Black individuals had a higher mortality risk than non-Hispanic White individuals that persisted in the fully adjusted model (AHR, 1.18; 95% CI, 1.07-1.29). The minimally adjusted model HR for mortality for Hispanic vs non-Hispanic White individuals was 1.15 (95% CI, 1.09-1.22), and the association was no longer present after full covariate adjustment (AHR, 0.98; 95% CI, 0.92-1.04).

**Table 2.** Racial and Ethnic Differences in Risk of CRC Mortality for Patients Younger Than 50 Years in the California Cancer Registry, 2000 to 2019

Race and ethnicity	No. of individuals	No. of CRC deaths	AHR (95% CI)		
			Minimally adjusted^a^	Base model^b^	Fully adjusted^c^
Asian American	3497	1039	1.06 (0.98-1.13)	0.99 (0.92-1.06)	0.99 (0.92-1.07)
Hispanic	6804	1998	1.15 (1.09-1.22)	1.15 (1.08-1.22)	0.98 (0.92-1.04)
Native Hawaiian or Other Pacific Islander	134	51	1.69 (1.27-2.23)	1.42 (1.07-1.87)	1.34 (1.01-1.76)
Non-Hispanic American Indian or Alaska Native	124	36	1.02 (0.74-1.39)	0.81 (0.59-1.12)	0.76 (0.55-1.06)
Non-Hispanic Black	1657	670	1.53 (1.40-1.66)	1.43 (1.30-1.56)	1.18 (1.07-1.29)
Non-Hispanic White	10 402	3089	1 [Reference]	1 [Reference]	1 [Reference]

Abbreviations: AHR, adjusted hazard ratio; CRC, colorectal cancer.

^a^ Adjusted for clustering by block group.

^b^ Adjusted for age at diagnosis and age at diagnosis squared, year of diagnosis and year of diagnosis squared, sex, tumor size, clustering by block group, and underlying stratification by American Joint Commission on Cancer (AJCC) stage, tumor grade, and tumor location.

^c^ Adjusted for age at diagnosis and age at diagnosis squared, year of diagnosis and year of diagnosis squared, sex, tumor size, marital status, insurance status, National Institute of Cancer–designated cancer center, neighborhood socioeconomic status, neighborhood urban-rural status, clustering by block group, and underlying stratification by AJCC stage, tumor grade, tumor location, and guideline-concordant treatment.

When disaggregating the Asian American groups by ethnic group compared with non-Hispanic White individuals (Table 3), the highest mortality likelihood was observed among Southeast Asian individuals (AHR, 1.29; 95% CI, 1.13-1.46) in the minimally adjusted model, which remained after adjusting for tumor characteristics (AHR, 1.17; 95% CI, 1.03-1.34) but not in the fully adjusted model (AHR, 1.10; 95% CI, 0.96-1.26).

**Table 3.** Racial and Ethnic Differences in Risk of CRC Mortality for Patients Younger Than 50 Years, Including Disaggregated Asian American Ethnic Groups, in the California Cancer Registry, 2000 to 2019

Race and ethnicity	No. of patients	No. of CRC deaths	AHR (95% CI)		
			Minimally adjusted^a^	Base model^b^	Fully adjusted^c^
Asian American					
Chinese	849	217	0.87 (0.76-0.99)	0.87 (0.75-1.00)	0.90 (0.78-1.04)
Filipino	864	264	1.10 (0.97-1.24)	0.95 (0.84-1.08)	0.95 (0.84-1.08)
Japanese	265	85	1.04 (0.84-1.30)	0.99 (0.80-1.23)	1.04 (0.83-1.29)
Korean	308	100	1.13 (0.93-1.37)	1.12 (0.92-1.37)	1.10 (0.90-1.35)
South Asian	274	72	1.01 (0.80-1.26)	0.92 (0.73-1.16)	0.97 (0.77-1.22)
Southeast Asian	699	245	1.29 (1.13-1.46)	1.17 (1.03-1.34)	1.10 (0.96-1.26)
Other Asian	238	56	0.92 (0.71-1.20)	0.92 (0.72-1.18)	0.93 (0.72-1.21)
Hispanic	6804	1998	1.15 (1.09-1.22)	1.15 (1.09-1.22)	0.98 (0.92-1.04)
Native Hawaiian or Other Pacific Islander	134	51	1.69 (1.27-2.23)	1.42 (1.07-1.87)	1.34 (1.01-1.77)
Non-Hispanic American Indian or Alaska Native	124	36	1.02 (0.74-1.39)	0.81 (0.59-1.13)	0.76 (0.55-1.06)
Non-Hispanic Black	1657	670	1.53 (1.41-1.66)	1.43 (1.30-1.56)	1.18 (1.08-1.29)
Non-Hispanic White	10 402	3089	1 [Reference]	1 [Reference]	1 [Reference]

Abbreviations: AHR, adjusted hazard ratio; CRC, colorectal cancer.

^a^ Adjusted for clustering by block group.

^b^ Adjusted for age at diagnosis and age at diagnosis squared, year of diagnosis and year of diagnosis squared, sex, tumor size, clustering by block group, and underlying stratification by American Joint Commission on Cancer (AJCC) stage, tumor grade, and tumor location.

^c^ Adjusted for age at diagnosis and age at diagnosis squared, year of diagnosis and year of diagnosis squared, sex, tumor size, marital status, insurance status, National Institute of Cancer–designated cancer center, neighborhood socioeconomic status, neighborhood urban-rural status, clustering by block group, and underlying stratification by AJCC stage, tumor grade, tumor location, and guideline-concordant treatment.

### Sequential Modeling Findings

**Figure 1.**
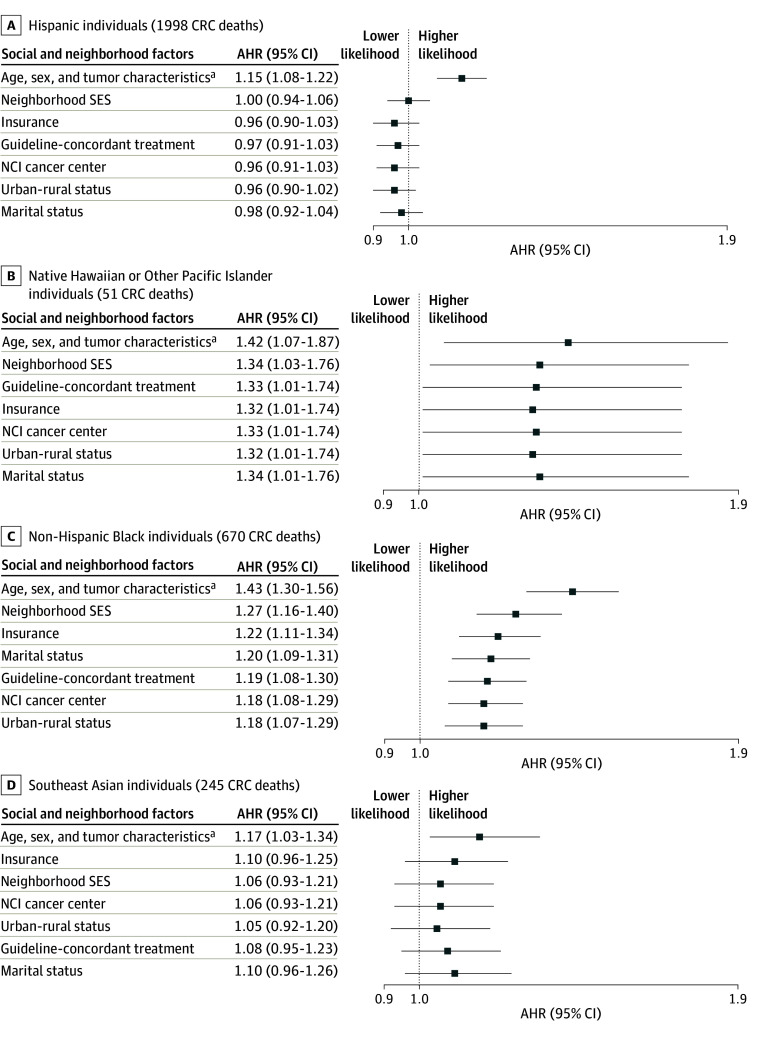
Adjusted Hazard Ratios (AHRs) for the Association of Race and Ethnicity With Risk of Colorectal Cancer (CRC)–Specific Mortality in Patients Younger Than 50 Years Social and neighborhood factors were added to the model one at a time based on their order of influence for that race and ethnicity. NCI indicates National Cancer Institute; SES, socioeconomic status. ^a^The base model was adjusted for age at diagnosis and age at diagnosis squared, year of diagnosis and year of diagnosis squared, sex, tumor size, and clustering by block group and underlying stratification by American Joint Committee on Cancer stage, tumor grade, and tumor location.

Sequential modeling was conducted to examine whether inclusion of additional factors (ordered from most decreased to most increased) attenuated EOCRC mortality effect estimates for Hispanic, Native Hawaiian or Other Pacific Islander, non-Hispanic Black, and Southeast Asian individuals compared with non-Hispanic White individuals (Figure 1 and eTable 2 in Supplement 1). The association with EOCRC mortality in Hispanic relative to non-Hispanic White individuals was not present after the addition of neighborhood SES to the model (AHR, 1.15 [95% CI, 1.08-1.22] in the base model; 1.00 [95% CI, 0.94-1.06] after adjustment for neighborhood SES). For Native Hawaiian or Other Pacific Islander compared to non-Hispanic White individuals, addition of neighborhood SES to the model led to a small attenuation in mortality likelihood (AHR, 1.42 [95% CI, 1.07-1.87] in the base model; AHR, 1.34 [95% CI, 1.03-1.76] after adjustment for neighborhood SES), but inclusion of additional variables did not further change mortality likelihood or account for the mortality difference. A similar likelihood was seen for mortality in non-Hispanic Black individuals compared with non-Hispanic White individuals, with an attenuation after addition of neighborhood SES (AHR, 1.43 [95% CI, 1.30-1.56] in the base model; AHR, 1.27 [95% CI, 1.16-1.40] after adjustment for neighborhood SES) and then small incremental contributions to the mortality difference for other variables, without fully accounting for the mortality difference. The association with EOCRC mortality in Southeast Asian relative to non-Hispanic White individuals was not present after the addition of insurance status to the model (AHR, 1.17 [95% CI, 1.03-1.34] in the base model; AHR, 1.10 [95% CI, 0.96-1.25] after adjustment for insurance status).

### Sensitivity Analyses

**Figure 2.**
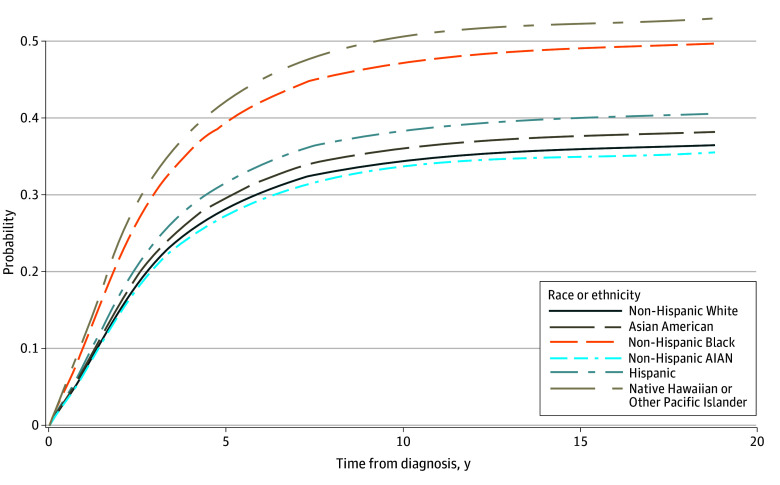
Cumulative Incidence Functions for Colorectal Cancer–Specific Mortality Controlling for the Competing Risk of Death From Other Causes in Patients Younger Than 50 Years AIAN indicates American Indian or Alaska Native.

Competing risk models of non–CRC-related death yielded numerically similar results, except for decreased EOCRC mortality likelihood among non-Hispanic American Indian or Alaska Native individuals (AHR, 0.70; 95% CI, 0.50-0.97) compared with non-Hispanic White individuals in the fully adjusted model (Figure 2 and eTables 3 and 4 in Supplement 1). All-cause mortality models yielded similar findings to the EOCRC-specific models (eTables 5 and 6 in Supplement 1). When stratifying our results by age groups (18-44 and 45-49 years), we observed differences in mortality likelihood for non-Hispanic Black adults, with higher EOCRC mortality likelihood in the younger but not the older group in the fully adjusted model (AHR, 1.30 [95% CI, 1.15-1.48] vs 1.05 [95% CI, 0.92-1.20]; *P* = .03 for interaction) (eTable 7 in Supplement 1). Differences by age group were also observed in the aggregated Asian American group.

## Discussion

In this population-based CCR cohort study, we observed a significantly higher EOCRC mortality likelihood among Native Hawaiian or Other Pacific Islander and non-Hispanic Black individuals compared with non-Hispanic White individuals, whereas the association disappeared among Hispanic individuals in the fully adjusted model. When disaggregating the Asian American group, heterogeneity in EOCRC mortality by ethnicity was evident. Notably, our findings differed by age group, particularly among Asian American and non-Hispanic Black individuals. The addition of neighborhood SES to the model showed the greatest attenuation in mortality likelihood in the Hispanic, Native Hawaiian or Other Pacific Islander, and non-Hispanic Black groups. As the CCR is one of the largest cancer registries in the US, these findings in California underscore disparities in EOCRC-related mortality that could exist and persist nationwide.

Few published reports on CRC-related mortality risk among disaggregated Asian American groups and for Native Hawaiian or Other Pacific Islander individuals exist. One study that broadly examined overall cancer survival disparities between Asian American and Native Hawaiian or Other Pacific Islander groups^18^ found survival was worse among Native Hawaiian or Other Pacific Islander adults compared with Asian American adults, justifying their disaggregation, which is consistent with our results for EOCRC. Neighborhood SES was associated with the largest change in mortality differences between Native Hawaiian or Other Pacific Islander and non-Hispanic White individuals, but the effect was modest and did not fully account for the mortality differences. Our results also show that although no significant difference in EOCRC-related mortality was observed between Asian American and non-Hispanic White adults, heterogeneity in mortality likelihood was shown with disaggregation, and the higher likelihood of mortality seen among Southeast Asian individuals was attributable to social and neighborhood factors. Recognizing the vast heterogeneity within the broad Asian American and Native Hawaiian or Other Pacific Islander categories,^19,20^ we aimed to disaggregate data for distinct ethnic groups. Our results show that the heterogeneity translated into different survival patterns across ethnic groups, reinforcing the need to examine these groups separately.^21,22^

Our findings of higher mortality likelihood among non-Hispanic Black individuals compared with non-Hispanic White individuals reinforce results of prior studies. Results of Surveillance, Epidemiology, and End Results (SEER)–based studies have shown higher CRC mortality risk in Black adults or significantly worse likelihood of survival compared with other racial and ethnic groups,^7,10,23^ similar to results reported in a National Cancer Database study.^24^ Additionally, examination of EOCRC incidence and survival trends shows that young Black adults have worse relative proximal or distal colon cancer survival, but no difference in relative rectal cancer survival, compared with White adults.^25^ We also found that neighborhood SES was associated with the largest attenuation in mortality risk between non-Hispanic Black and non-Hispanic White individuals, although this, along with the small incremental differences seen with the addition other social and clinical factors, does not fully account for the differences.

A growing body of evidence shows that Hispanic individuals have rapidly rising EOCRC rates, suggesting that more needs to be done to address this burden in this large ethnic group in the US.^26^ In the present study, Hispanic individuals with EOCRC had increased CRC-related mortality risk compared with non-Hispanic White individuals, a difference that persisted when adjusting for tumor characteristics, but attenuated after additional adjustment for social and neighborhood contextual factors, which were found to be associated with the largest attenuation in mortality differences. The mortality findings are supported by published reports showing lower 5-year survival among young Hispanic individuals compared with non-Hispanic White young adults.^7,24^

Using methodology from a prior study of CCR data,^27^ we found that neighborhood SES was associated with attenuation in EOCRC-related racial and ethnic mortality differences. Prior studies have shown that lower neighborhood-level SES, in general, is associated with worse survival outcomes in adults of any age with CRC.^28,29^ Similar to our study, Aloysius et al^23^ used SEER data linked with the American Community Survey and found that poverty, unemployment, and lower income were associated with worse EOCRC-related survival across various racial and ethnic groups, while higher educational attainment and access to commercial health insurance were associated with improved survival. Measures of neighborhood SES, deprivation, and poverty reflect the social and economic composition of its residents and have been shown to be robust indicators of specific social and built environmental attributes that determine the level of access to high-quality goods and services (including health care) and exposures to harmful pollutants and stressors.^30,31,32^ Our findings add to this body of evidence by disaggregating the Asian American categories and highlighting additional social determinants of health, including neighborhood SES, which could contribute to EOCRC mortality differences across racial and ethnic groups. These findings could drive tailored interventions, including targeted education and resources to the most impacted communities to pursue preventive care, such as screening. The particularly robust changes in association between race and ethnicity and mortality likelihood when adjusting for neighborhood SES among Hispanic, Native Hawaiian or Other Pacific Islander, and non-Hispanic Black individuals highlight the need to unpack other neighborhood-level mechanisms that could be contributing to disparate EOCRC mortality burdens. Given the pronounced increase in young and middle-aged adults with CRC alongside decreasing rates in older individuals,^2^ these results underscore how CRC is now impacting individuals in the prime of their life cycle in relation to family, career, and productivity.

### Limitations

Our study has some limitations. In using recently available cancer registry data, we were limited by the relatively short length of follow-up. The total sample sizes and number of CRC deaths in some racial and ethnic groups were low, which could lead to imprecise AHR estimates. Of note, prior studies have found cancer-based survival studies might miss deaths among Hispanic and Asian adults, which could cause underestimation of the mortality risk.^33^ Limitations inherent to cancer registry data, such as misclassification of race and ethnicity, could have impacted our results; however, prior research has shown this to be minimal,^21,34^ especially since classification of Hispanic ethnicity and Asian American and Native Hawaiian or Other Pacific Islander races use existing validated algorithms.^14^ Although we were able to conduct disaggregated analysis within the Asian American group, we did not disaggregate the Hispanic group, given the lack of known Hispanic origin among over 40% of Hispanic cancer cases in SEER cancer registry databases, and the well-documented limited heterogeneity in this ethnic group in California (approximately 80% Mexican descent). The generalizability of our study to the broader US population is limited; studies extending beyond California are needed to assess heterogeneity. While we measured numerous sociodemographic variables at the individual level, neighborhood SES and urban-rural status were the only aggregate-level variables available to link to the data, limiting further investigation of the potential impact of neighborhood-level factors on CRC death. Additionally, our ability to further examine potential associations between neighborhood SES and other factors, which could disentangle potential disparity mechanisms, was limited by small sample sizes.

## Conclusions

Results of our cohort study provide evidence of racial and ethnic disparities in EOCRC mortality, particularly among Native Hawaiian or Other Pacific Islander and non-Hispanic Black patients, and heterogeneity in risk when disaggregating the Asian American racial group. Examination of potential factors associated with differences in mortality risk showed that neighborhood SES was associated with the largest attenuation in EOCRC-related disparities among Native Hawaiian or Other Pacific Islander, non-Hispanic Black, and Southeast Asian individuals. These findings provide novel and important data underscoring the role of social determinants of health in EOCRC-related mortality and the need to address barriers to care to ensure greater equity in survival for a cancer that affects individuals whose lives are cut short in their prime.

## Notes

Supplement 1. **eMethods.** Data Acquisition and Analysis **eTable 1.** Guideline-Concordant Treatment Defined Based on Hines **eTable 2.** Order of Influence of Social and Neighborhood Factors on Colorectal Cancer Mortality Risk by Racial and Ethnic Group, California, 2000 to 2019 **eTable 3.** Racial and Ethnic Differences in Risk of Colorectal Cancer Mortality for Patients Younger Than 50 Years Using Fine and Gray Competing Risks Estimation, California, 2000 to 2019 **eTable 4.** Racial and Ethnic Differences in Risk of Colorectal Cancer Mortality Including Disaggregated Asian American Groups Using Fine and Gray Competing Risks Estimation, California, 2000 to 2019 **eTable 5.** Racial and Ethnic Differences in Risk of All-Cause Mortality for Patients Younger Than 50 Years, California, 2000 to 2019 **eTable 6.** Racial and Ethnic Differences in Risk of All-Cause Mortality Including Disaggregated Asian American Groups, California, 2000 to 2019 **eTable 7.** Racial and Ethnic Differences in Risk of Colorectal Cancer Mortality for Patients Aged 18-44 vs 45-49 Years, California, 2000 to 2019

Supplement 2.
Data Sharing Statement
