# Supplementary material for: Racial and Ethnic Variation in Survival in Early-Onset Colorectal Cancer
Source: JAMA Netw Open. 2024 Nov 22;7(11):e2446820. doi: 10.1001/jamanetworkopen.2024.46820 (PMC11584933; doi:10.1001/jamanetworkopen.2024.46820)
Supplement: Supplement 1. — eMethods. Data Acquisition and Analysis eTable 1. Guideline-Concordant Treatment Defined Based on Hines eTable 2. Order of Influence of Social and Neighborhood Factors on Colorectal Cancer Mortality Risk by Racial and Ethnic Group, California, 2000 to 2019 eTable 3. Racial and Ethnic Differences in Risk of Colorectal Cancer Mortality for Patients Younger Than 50 Years Using Fine and Gray Competing Risks Estimation, California, 2000 to 2019 eTable 4. Racial and Ethnic Differences in Risk of Colorectal Cancer Mortality Including Disaggregated Asian American Groups Using Fine and Gray Competing Risks Estimation, California, 2000 to 2019 eTable 5. Racial and Ethnic Differences in Risk of All-Cause Mortality for Patients Younger Than 50 Years, California, 2000 to 2019 eTable 6. Racial and Ethnic Differences in Risk of All-Cause Mortality Including Disaggregated Asian American Groups, California, 2000 to 2019 eTable 7. Racial and Ethnic Differences in Risk of Colorectal Cancer Mortality for Patients Aged 18-44 vs 45-49 Years, California, 2000 to 2019 [file jamanetwopen-e2446820-s001.pdf]

Supplementary Online Content

Demb J, Gomez S, Canchola AJ, et al. Racial and ethnic variation in early-onset colorectal cancer survival in California Cancer Registry. *JAMA Netw Open*. 2024;7(11):e2446820. doi:10.1001/jamanetworkopen.2024.46820

eMethods. Data Acquisition and Analysis

eTable 1. Guideline-Concordant Treatment Defined Based on Hines

eTable 2. Order of Influence of Social and Neighborhood Factors on Colorectal Cancer Mortality Risk by Racial and Ethnic Group, California, 2000 to 2019

eTable 3. Racial and Ethnic Differences in Risk of Colorectal Cancer Mortality for Patients Younger Than 50 Years Using Fine and Gray Competing Risks Estimation, California, 2000 to 2019

eTable 4. Racial and Ethnic Differences in Risk of Colorectal Cancer Mortality Including Disaggregated Asian American Groups Using Fine and Gray Competing Risks Estimation, California, 2000 to 2019

eTable 5. Racial and Ethnic Differences in Risk of All-Cause Mortality for Patients Younger Than 50 Years, California, 2000 to 2019

eTable 6. Racial and Ethnic Differences in Risk of All-Cause Mortality Including Disaggregated Asian American Groups, California, 2000 to 2019

eTable 7. Racial and Ethnic Differences in Risk of Colorectal Cancer Mortality for Patients Aged 18-44 vs 45-49 Years, California, 2000 to 2019

This supplementary material has been provided by the authors to give readers additional information about their work.

© 2024 Demb J et al. *JAMA Network Open.*

eMethods. Data Acquisition and Analysis

Study Participant Exclusions

Patients were excluded from the analytic sample hierarchically as follows: diagnosis from death certificate or autopsy only (n=58); diagnosis not microscopically confirmed (n=181); histology not adenocarcinoma (n=2,844; adenocarcinoma histologic codes included in analysis: 8140, 8141, 8143, 8144, 8210, 8211, 8213, 8220, 8221, 8255, 8260-8265, 8310, 8323, 8440, 8460, 8470, 8472, 8480-8482, 8570, 8574, and 8576); no follow-up (n=55); residential address uncertain or not geocodable (n=1,032); or race and ethnicity unknown (n=163). This resulted in a study population that included 22,834 individuals.

Data Acquisition

Patient’s insurance status was based on primary and secondary sources of payment on the last admission for initial diagnosis and/or treatment. We classified insurance status according to six hierarchical categories: No insurance/self-pay, Medicaid only, any Medicare, any military/other public insurance, any private insurance and unknown insurance status. Treatment variables utilized were based on the first course of treatment and the dates when treatments were initiated. These included surgery, chemotherapy, and radiation, coded as no, yes, and unknown. Guideline concordant treatment was defined as follows: for colon cancer: AJCC stage I-II: surgery; stage III: surgery and adjuvant chemotherapy; stage IV: chemotherapy; for rectal cancer: stage I: surgery; stage II-III: surgery, chemotherapy, and radiation; stage IV: chemotherapy (Supplemental Table 1).[16] Concordance was defined as receiving the minimal treatment, i.e., overtreatment was classified as concordant. Adjuvant chemotherapy was defined as chemotherapy within 1 year after surgery based on treatment dates. These guidelines from Hines et al. were used as they corresponded to the diagnosis years included in the analysis. Residential urban/rural status was defined based on 2000 U.S. Census (for cases diagnosed in

© 2024 Demb J et al. *JAMA Network Open.*

2005 and earlier) and 2010 U.S. Census data (for cases diagnosed in 2006 and forward) on Urbanized Areas and Urban Clusters.

Statistical Analysis

The proportional hazards assumption was tested by examining the correlation between time and scaled Schoenfeld residuals for all variables. Variables that violated the proportional hazards assumption were included in the model as an underlying stratification factor, which allowed the baseline hazard to vary by the levels of these factors. A sensitivity analysis was conducted using the Fine and Gray method to additionally account for the potential competing risk of non-CRC-related death in the Cox regression analysis.[19] All models were adjusted for clustering by census block group using a sandwich estimator of the covariance structure that accounts for intracluster dependence.

To assess the relative contribution of the social and neighborhood factors to risk of EOCRC mortality in specific races and ethnicities after accounting for age, sex, and tumor characteristics in a base model, sequential models were run, where the social and neighborhood factors were entered into the model one at a time based on their level of influence. To determine the level of influence of each variable, we ran separate models adding in each candidate social and neighborhood factor and clinical factor alone to the base model, and computed the percent change to the parameter estimate for each race and ethnicity (compared to NH White) with the addition of that variable. Then, separately for each race and ethnicity, variables were put in order of their level of influence by sorting the percent change to the parameter estimate from most decreased to most increased. Supplemental Table 2 shows the level of influence for the different factors sorted by the percent change to the parameter estimate for each racial and ethnic group in the primary analysis.

© 2024 Demb J et al. *JAMA Network Open.*

eTable 1. Guideline-Concordant Treatment Defined Based on Hines

Colon Cancer	Hines 2015	Used
Stage I-II	Surgery with curative intent	Surgery
Stage III (AJCC)	Surgery followed by adjuvant chemo	Surgery and adjuvant chemotherapy*
Stage IV	Systemic chemotherapy	Chemotherapy
Rectal Cancer		
Stage I	Surgery with curative intent	Surgery
Stages II-III (AJCC) regional or regional with lymph node involvement	Surgery and both radiation and chemo (neoadjuvant or adjuvant)	Surgery and chemotherapy and radiation
Stage IV	Systemic chemotherapy	Chemotherapy

The Hines paper defined minimal treatment received as concordant (i.e., overtreatment was classified as concordant).
*Adjuvant chemotherapy defined as chemotherapy within 1 year after surgery based on treatment dates.
Ref: Hines RB, Barrett A, Twumasi-Ankrah P, et al. Predictors of guideline treatment nonadherence and the impact on survival in patients with colorectal cancer. *J Natl Compr Canc Netw*. 2015;13(1):51-60. doi:10.6004/jnccn.2015.0008

© 2024 Demb J et al. *JAMA Network Open.*

eTable 2. Order of Influence of Social and Neighborhood Factors on Colorectal Cancer Mortality Risk by Racial and Ethnic Group, California, 2000 to 2019

Racial or Ethnic Group	*Base Model Parameter Estimate**	*Adjusted Model Parameter Estimate***	*Difference between Adjusted and Baseline*	*Percent Change*	*Factors*
Hispanic	0.13886	-0.00388	-0.14274	-1.02795	nSES
	0.13886	0.04796	-0.09090	-0.65461	Insurance
	0.13886	0.12250	-0.01636	-0.11784	Guideline treatment
	0.13886	0.12639	-0.01247	-0.08983	NCI Cancer Center
	0.13886	0.12835	-0.01051	-0.07571	Urban/Rural
	0.13886	0.13835	-0.00051	-0.00370	Marital Status
NH Black	0.35454	0.24288	-0.11166	-0.31493	nSES
	0.35454	0.26260	-0.09194	-0.25932	Insurance
	0.35454	0.30923	-0.04531	-0.12781	Marital Status
	0.35454	0.32920	-0.02534	-0.07148	Guideline treatment
	0.35454	0.33878	-0.01576	-0.04446	NCI Cancer Center
	0.35454	0.34952	-0.00502	-0.01417	Urban/Rural
NHPI	0.34778	0.29541	-0.05237	-0.15058	nSES
	0.34778	0.33004	-0.01774	-0.05100	Guideline treatment
	0.34778	0.33037	-0.01741	-0.05005	Insurance

Racial or Ethnic Group	*Base Model Parameter Estimate**	*Adjusted Model Parameter Estimate***	*Difference between Adjusted and Baseline*	*Percent Change*	*Factors*
	0.34778	0.34663	-0.00115	-0.00329	NCI Cancer Center
	0.34778	0.34875	0.00097	0.00278	Urban/Rural
	0.34778	0.35433	0.00655	0.01884	Marital Status
Southeast Asian	0.16077	0.09465	-0.06612	-0.41127	Insurance
	0.16077	0.09770	-0.06307	-0.39229	nSES
	0.16077	0.15334	-0.00743	-0.04621	NCI Cancer Center
	0.16077	0.16690	0.00613	0.03813	Urban/Rural
	0.16077	0.17066	0.00989	0.06154	Guideline treatment
	0.16077	0.17532	0.01456	0.09054	Marital Status

*Parameter estimate for the specific race and ethnicity compared to NHW in the base model.

**Each factor added to the base model individually.

© 2024 Demb J et al. *JAMA Network Open.*

eTable 3. Racial and Ethnic Differences in Risk of Colorectal Cancer Mortality for Patients Younger Than 50 Years Using Fine and Gray Competing Risks Estimation, California, 2000 to 2019

Race/ethnicity	*No. CRC Deaths*	*No. Other Deaths*	*Hazard Ratio** (95% Confidence Interval)*	*Hazard Ratio*** (95% Confidence Interval)*	*Hazard Ratio**** (95% Confidence Interval)*
Asian American	1039	111	1.06 (0.99-1.14)	1.00 (0.93-1.07)	1.00 (0.93-1.07)
Hispanic	1998	274	1.15 (1.08-1.21)	1.12 (1.06-1.19)	0.97 (0.91-1.04)
NH AIAN	36	15	0.97 (0.70-1.33)	0.75 (0.54-1.05)	0.70 (0.50-0.97)
NH Black	670	84	1.51 (1.39-1.65)	1.40 (1.28-1.53)	1.17 (1.07-1.29)
NH White	3089	487	1.00 (Referent)	1.00 (Referent)	1.00 (Referent)
NHPI	51	8	1.66 (1.25-2.20)	1.42 (1.08-1.87)	1.34 (1.02-1.77)

*The minimally adjusted model was adjusted for clustering by block group.
**The base model was adjusted for age and age squared, year of diagnosis and year of diagnosis squared, sex, tumor size, and clustering by block group; and had underlying stratification by AJCC stage, tumor grade, and tumor location.
***The fully adjusted model was adjusted for age and age squared, year of diagnosis and year of diagnosis squared, sex, tumor size, marital status, insurance status, NCI-designated cancer center, neighborhood SES, neighborhood urban/rural status, and clustering by block group; and had underlying stratification by AJCC stage, tumor grade, tumor location, and guideline concordant treatment.
NH AIAN=Non-Hispanic American Indian or Alaska Native; NH Black=Non-Hispanic Black; NH White=Non-Hispanic White; NHPI=Native Hawaiian or Pacific Islander.

© 2024 Demb J et al. *JAMA Network Open.*

eTable 4. Racial and Ethnic Differences in Risk of Colorectal Cancer Mortality Including Disaggregated Asian American Groups Using Fine and Gray Competing Risks Estimation, California, 2000 to 2019

Race and ethnicity	*No. CRC Deaths*	*No. Other Deaths*	*Minimally Adjusted Hazard Ratio* (95% Confidence Interval)*	*Base Model Hazard Ratio** (95% Confidence Interval)*	*Fully Adjusted Hazard Ratio*** (95% Confidence Interval)*
Chinese	217	20	0.88 (0.77-1.01)	0.90 (0.78-1.03)	0.93 (0.81-1.07)
Filipino	264	31	1.10 (0.97-1.25)	0.96 (0.85-1.08)	0.95 (0.84-1.07)
Hispanic	1998	274	1.15 (1.08-1.21)	1.13 (1.06-1.19)	0.97 (0.91-1.04)
Japanese	85	5	1.07 (0.86-1.33)	1.04 (0.85-1.28)	1.08 (0.88-1.33)
Korean	100	13	1.13 (0.93-1.37)	1.08 (0.88-1.31)	1.07 (0.88-1.30)
NH AIAN	36	15	0.97 (0.70-1.33)	0.75 (0.54-1.05)	0.70 (0.50-0.97)
NH Black	670	84	1.51 (1.39-1.65)	1.40 (1.28-1.53)	1.18 (1.07-1.29)
NH White	3089	487	1.00 (Referent)	1.00 (Referent)	1.00 (Referent)
NHPI	51	8	1.66 (1.25-2.20)	1.42 (1.08-1.87)	1.34 (1.02-1.77)
Other Asian	56	9	0.91 (0.70-1.18)	0.93 (0.72-1.19)	0.93 (0.72-1.21)
South Asian	72	<5	1.02 (0.82-1.28)	0.96 (0.77-1.20)	1.00 (0.80-1.26)
Southeast Asian	245	30	1.28 (1.12-1.45)	1.15 (1.01-1.31)	1.08 (0.95-1.24)

*The minimally adjusted model was adjusted for clustering by block group.
**The base model was adjusted for age and age squared, year of diagnosis and year of diagnosis squared, sex, tumor size, and clustering by block group; and had underlying stratification by AJCC stage, tumor grade, and tumor location.
***The fully adjusted model was adjusted for age and age squared, year of diagnosis and year of diagnosis squared, sex, tumor size, marital status, insurance status, NCI-designated cancer center, neighborhood SES, neighborhood urban/rural status, and clustering by block group; and had underlying stratification by AJCC stage, tumor grade, tumor location, and guideline concordant treatment.
NH AIAN=Non-Hispanic American Indian or Alaska Native; NH Black=Non-Hispanic Black; NH White=Non-Hispanic White; NHPI=Native Hawaiian or Pacific Islander.

© 2024 Demb J et al. *JAMA Network Open.*

eTable 5. Racial and Ethnic Differences in Risk of All-Cause Mortality for Patients Younger Than 50 Years, California, 2000 to 2019

Race/ethnicity	*No. Deaths*	*No. Alive*	*Hazard Ratio* (95% Confidence Interval)*	*Hazard Ratio** (95% Confidence Interval)*	*Hazard Ratio*** (95% Confidence Interval)*
Asian American	1197	2347	1.03 (0.96-1.10)	0.98 (0.92-1.05)	0.98 (0.91-1.05)
Hispanic	2357	4532	1.16 (1.10-1.22)	1.16 (1.10-1.22)	0.96 (0.91-1.02)
NH AIAN	52	73	1.26 (0.97-1.64)	1.03 (0.77-1.36)	0.95 (0.71-1.26)
NH Black	765	903	1.48 (1.37-1.61)	1.39 (1.28-1.51)	1.12 (1.03-1.22)
NH White	3647	6826	1.00 (Referent)	1.00 (Referent)	1.00 (Referent)
NHPI	60	75	1.72 (1.33-2.21)	1.48 (1.14-1.91)	1.39 (1.07-1.81)

*The minimally adjusted model was adjusted for clustering by block group.
**The base model was adjusted for age and age squared, year of diagnosis and year of diagnosis squared, sex, tumor size, and clustering by block group; and had underlying stratification by AJCC stage, tumor grade, and tumor location.
***The fully adjusted model was adjusted for age and age squared, year of diagnosis and year of diagnosis squared, sex, tumor size, marital status, insurance status, NCI-designated cancer center, neighborhood SES, neighborhood urban/rural status, and clustering by block group; and had underlying stratification by AJCC stage, tumor grade, tumor location, and guideline concordant treatment.
NH AIAN=Non-Hispanic American Indian or Alaska Native; NH Black=Non-Hispanic Black; NH White=Non-Hispanic White; NHPI=Native Hawaiian or Pacific Islander.

© 2024 Demb J et al. *JAMA Network Open.*

eTable 6. Racial and Ethnic Differences in Risk of All-Cause Mortality Including Disaggregated Asian American Groups, California, 2000 to 2019

Race and ethnicity	*No. Deaths*	*No. Alive*	*Minimally Adjusted Hazard Ratio* (95% Confidence Interval)*	*Base Model Hazard Ratio** (95% Confidence Interval)*	*Fully Adjusted Hazard Ratio*** (95% Confidence Interval)*
Chinese	249	612	0.84 (0.74-0.95)	0.84 (0.74-0.95)	0.87 (0.76-0.99)
Filipino	309	569	1.08 (0.96-1.22)	0.97 (0.87-1.09)	0.98 (0.87-1.10)
Hispanic	2357	4532	1.16 (1.10-1.22)	1.16 (1.10-1.22)	0.96 (0.91-1.02)
Japanese	92	175	0.95 (0.77-1.18)	0.94 (0.76-1.15)	0.98 (0.80-1.21)
Korean	119	195	1.13 (0.94-1.34)	1.13 (0.94-1.36)	1.09 (0.90-1.32)
NH AIAN	52	73	1.26 (0.97-1.64)	1.03 (0.77-1.36)	0.95 (0.71-1.26)
NH Black	765	903	1.48 (1.37-1.61)	1.40 (1.29-1.52)	1.12 (1.03-1.22)
NH White	3647	6826	1.00 (Referent)	1.00 (Referent)	1.00 (Referent)
NHPI	60	75	1.72 (1.33-2.21)	1.48 (1.14-1.91)	1.39 (1.07-1.81)
Other Asian	69	173	0.96 (0.76-1.22)	0.97 (0.77-1.22)	0.98 (0.77-1.24)
South Asian	79	199	0.94 (0.76-1.17)	0.86 (0.69-1.07)	0.93 (0.75-1.15)
Southeast Asian	280	424	1.26 (1.11-1.42)	1.16 (1.03-1.31)	1.07 (0.95-1.22)

*The minimally adjusted model was adjusted for clustering by block group.
**The base model was adjusted for age and age squared, year of diagnosis and year of diagnosis squared, sex, tumor size, and clustering by block group; and had underlying stratification by AJCC stage, tumor grade, and tumor location.
***The fully adjusted model was adjusted for age and age squared, year of diagnosis and year of diagnosis squared, sex, tumor size, marital status, insurance status, NCI-designated cancer center, neighborhood SES, neighborhood urban/rural status, and clustering by block group; and had underlying stratification by AJCC stage, tumor grade, tumor location, and guideline concordant treatment.
NH AIAN=Non-Hispanic American Indian or Alaska Native; NH Black=Non-Hispanic Black; NH White=Non-Hispanic White; NHPI=Native Hawaiian or Pacific Islander.

© 2024 Demb J et al. *JAMA Network Open.*

eTable 7. Racial and Ethnic Differences in Risk of Colorectal Cancer Mortality for Patients Aged 18-44 vs 45-49 Years, California, 2000 to 2019

Race/ethnicity	*No. CRC Deaths*	*No. Alive*	*Hazard Ratio* (95% Confidence Interval)*	*Hazard Ratio** (95% Confidence Interval)*	*Hazard Ratio*** (95% Confidence Interval)*
**Ages 18-44**					
Asian American	576	1249	1.17 (1.06 -1.29)	1.08 (0.98-1.18)	1.10 (1.00-1.21)
Hispanic	1163	2835	1.17 (1.09-1.27)	1.17 (1.08-1.27)	1.01 (0.92-1.10)
NH AIAN	19	53	0.91 (0.59-1.40)	0.86 (0.55-1.36)	0.81 (0.51-1.28)
NH Black	318	491	1.50 (1.33-1.70)	1.49 (1.31-1.70)	1.30 (1.15-1.48)
NH White	1476	3632	1.00 (Referent)	1.00 (Referent)	1.00 (Referent)
NHPI	29	48	1.77 (1.23-2.57)	1.31 (0.90-1.91)	1.25 (0.86-1.81)
**Ages 45-49**					
Asian American	463	1209	0.94 (0.85-1.05)	0.91 (0.82-1.01)	0.90 (0.81-1.00)
Hispanic	835	1971	1.14 (1.05-1.24)	1.12 (1.03-1.22)	0.94 (0.86-1.04)
NH AIAN	17	35	1.19 (0.75-1.88)	0.75 (0.47-1.19)	0.70 (0.44-1.11)
NH Black	352	496	1.55 (1.38-1.74)	1.36 (1.20-1.54)	1.05 (0.92-1.20)
NH White	1613	3681	1.00 (Referent)	1.00 (Referent)	1.00 (Referent)
NHPI	22	35	1.59 (1.03-2.46)	1.58 (1.04-2.38)	1.38 (0.87-2.18)

© 2024 Demb J et al. *JAMA Network Open.*

Race/ethnicity	*No. CRC Deaths*	*No. Alive*	*Hazard Ratio* (95% Confidence Interval)*	*Hazard Ratio** (95% Confidence Interval)*	*Hazard Ratio*** (95% Confidence Interval)*

*The minimally adjusted model was adjusted for clustering by block group.
**The base model was adjusted for age and age squared, year of diagnosis and year of diagnosis squared, sex, tumor size, and clustering by block group; and had underlying stratification by AJCC stage, tumor grade, and tumor location.
***The fully adjusted model was adjusted for age and age squared, year of diagnosis and year of diagnosis squared, sex, tumor size, marital status, insurance status, NCI-designated cancer center, neighborhood SES, neighborhood urban/rural status, and clustering by block group; and had underlying stratification by AJCC stage, tumor grade, tumor location, and guideline concordant treatment.
NH AIAN=Non-Hispanic American Indian or Alaska Native; NH Black=Non-Hispanic Black; NH White=Non-Hispanic White; NHPI=Native Hawaiian or Pacific Islander.
Global p-interaction between race/ethnicity and age in fully adjusted model p=0.06. Individual p-interactions: Asian (p<0.01); Hispanic (p=0.35); NH AIAN (p=0.64); NH Black (p=0.03); NHPI (p=0.95).

© 2024 Demb J et al. *JAMA Network Open.*
