# Supplementary material for: Racial and Ethnic Variation in Survival in Early-Onset Colorectal Cancer
Source: JAMA Netw Open. 2024 Nov 22;7(11):e2446820. doi: 10.1001/jamanetworkopen.2024.46820 (PMC11584933; doi:10.1001/jamanetworkopen.2024.46820)
Supplement: Supplement 2. — Data Sharing Statement [file jamanetwopen-e2446820-s002.pdf]

Data Sharing Statement

Demb. Racial and Ethnic Variation in Survival in Early-Onset Colorectal Cancer. *JAMA Netw Open*. Published November 22, 2024. doi:10.1001/jamanetworkopen.2024.46820

Data
Data available: No

Additional Information
Explanation for why data not available: Data from California Cancer Registry are protected and only accessible via an approved data access request. Thus, we will not be making these data available outside of this structure.
